# The Characteristics and Function of S100A7 Induction in Squamous Cell Carcinoma: Heterogeneity, Promotion of Cell Proliferation and Suppression of Differentiation

**DOI:** 10.1371/journal.pone.0128887

**Published:** 2015-06-08

**Authors:** Zhi Qi, Ting Li, Fei Kong, Yunguang Li, Rui Wang, Junhao Wang, Qianqian Xiao, Weiqing Zhang, Suozhu Sun, Dacheng He, Xueyuan Xiao

**Affiliations:** 1 Key laboratory of Cell Proliferation and Regulation of Ministry of Education, Beijing Normal University, Beijing, China; 2 Universities’ Confederated Institute of Proteomics, Beijing, China; 3 Department of Pathology, General Hospital of the Second Artillery, Beijing, China; University of Central Florida, UNITED STATES

## Abstract

S100A7 is highly expressed in squamous cell carcinomas (SCC) and is related to the terminal differentiation of keratinocytes. However, its characteristic and function in SCC is not very known. In this present study, we used immunohistochemistry to examine the expression of S100A7 in 452 SCC specimens, including the lung, esophagus, oral cavity, skin, cervix, bladder, and three SCC cell lines. We found that S100A7-positive staining showed significant heterogeneity in six types of SCC specimen and three SCC cell lines. Further examination found that S100A7-positive cells and its expression at mRNA and protein levels could be induced in HCC94, FaDu, and A-431 cells both in vitro and in vivo using immunohistochemistry, real-time PCR, and Western blotting. Notably, the upregulation of squamous differentiation markers, including keratin-4, keratin-13, TG-1, and involucrin, also accompanied S100A7 induction, and a similar staining pattern of S100A7 and keratin-13 was found in HCC94 cells both in vitro and in vivo. Further study revealed that the overexpression of S100A7 significantly increased proliferation and inhibited squamous differentiation in A-431 cells both in vitro and in vivo. Conversely, silencing S100A7 inhibited cell growth and survival and increased the expression of keratin-4, keratin-13, TG-1, and involucrin in HCC94 cells. Therefore, these results demonstrate that S100A7 displays heterogeneous and inducible characteristic in SCC and also provide novel evidence that S100A7 acts as a dual regulator in promoting proliferation and suppressing squamous differentiation of SCC.

## Introduction

Squamous cell carcinomas (SCCs) are the most common cancer and can be very aggressive and metastatic. SCC shows deregulation and defects in cell differentiation [1–2], and these defects are hypothesized to help squamous cells survive and escape terminal differentiation. Despite operation, radiotherapy, and chemotherapy, SCC lesions often recur and spread to other body sites, such as the lungs. Therefore, it is important to identify the molecules that inhibit the aberrant proliferation of SCC and simultaneously reinstate a normal differentiation program. This strategy may be an additional useful strategy for the clinical treatment of SCCs.

S100A7 (psoriasin) belongs to the S100 multigenic family of calcium-modulated proteins of the EF-hand type and was originally identified in psoriatic keratinocytes [3–4]. In addition to its antibacterial effects [5], S100A7 expression is up-regulated in breast cancer and many types of squamous cell carcinomas, including lung, oral cavity, bladder, and skin, and also plays an important role in carcinogenesis and metastasis [6–18]. Several studies report that the high level of S100A7 expression is always observed in highly differentiated SCCs, and weak or loss of expression is observed in moderately or poorly-differentiated SCCs [7,11,12,18], suggesting a specific association of S100A7 expression with SCC proliferation and differentiation. The involvement of S100A7 in the differentiation process is also suggested by the fact that S100A7 is located within a gene cluster in chromosome 1q21, the epidermal differentiation complex. This cluster also contains epidermal differentiation markers, such as several cytokeratins and involucrin [19]. Intriguingly, the level of S100A7 expression in SCC tissues is inconsistent with that in SCC cell line cultured in vitro. Because S100A7 expression is relatively low or undetected in SCC cells in vitro; however, it has been reported that S100A7 is induced in keratinocytes by certain stimuli, such as suspension and confluent culture [19]. Thinking along the connection between S100A7 expression in vivo, in vitro, and induction, we asked: can S100A7 be induced in SCC cell lines similar as keratinocytes? If so, what is the function of S100A7 in SCC cells? In the present study, we found that S100A7-positive staining showed significant heterogeneity in six types of SCC specimen and three SCC cell lines. Further examination found that S100A7-positive cells could be induced in HCC94, FaDu, and A-431 cells both in vitro and in vivo. Notably, the upregulation of squamous differentiation markers, including keratin-4, keratin-13, TG-1, and involucrin, also accompanied S100A7 induction, and a similar staining pattern of S100A7 and keratin-13 was found in HCC94 cells both in vitro and in vivo. Further study revealed that the overexpression of S100A7 significantly increased proliferation and inhibited squamous differentiation in A-431 cells both in vitro and in vivo. Conversely, knockdown S100A7 inhibited cell growth and survival and increased the expression of keratin-4, keratin-13, TG-1, and involucrin in HCC94 cells. Overall, our findings provide novel evidence that S100A7 acts as a dual regulator in promoting proliferation and suppressing squamous differentiation of SCC.

## Materials and Methods

### Cell lines and culture conditions

The human carcinoma cell lines A-431, HCC94, and FaDu were purchased from the Chinese Academy of Sciences Committee Type Culture Collection Cell Bank and the cell lines were authenticated by short tandem repeat analysis at HK Gene Science Technology Co (Beijing, China). All cells were cultured in accordance with the corresponding culture method of the ATCC. Cultured cells that reached∼60% density were defined as pre-confluent. For the confluent induction experiment, the cells were maintained at confluence for different numbers of days and the medium was routinely changed each day. Suspension culture was achieved by plating the cells in Poly-HEMA coated (12mg/ml dissolved in 95% ethanol) 6-well and 96-well plates in medium with 10% fetal bovine serum for different numbers of days.

### Tissue specimens

Four hundred and fifty-two SCC tissue specimens were obtained from thirteen cancer tissue arrays. One lung SCC tissue array (No Hlug-squ150CS-01), one esophageal SCC tissue array (No HEso-squ172Sur-01), and one bladder cancer tissue array (No OD-CT-UrBla03-003) were purchased from Shanghai Outdo Biotech Co., Ltd,. One oral cavity SCC tissue array (OR601a), three skin cancer tissue arrays (SK801b array, SK802a and SK483), one cervical SCC tissue array (CR803), and six cancer tissue arrays (No CC04-01-001, T271a,T022c, T124b, CR803 and SK483) including normal tissues of lung, tong, esophagus, bladder, cervix and skin were purchased from Xi An Alenabio Co. All cancer tissues were obtained from surgically treated patients who gave informed consent and the Ethics Committee of the General Hospital of the Second Artillery (No. KY2013024) approved this protocol. All participants provide their written informed consent to participate in this study. All methods were carried out in accordance with the approved protocol by above Ethics Committee. All cancer patients received a pathological diagnosis, and none had received prior therapy.

### Reverse transcription and Real-time PCR

Total RNA was extracted from the cells for the generation of single-stranded cDNA. The real-time PCR was performed using an ABI 7300 Real-time PCR System with the Power SYBR Green PCR Master Mix in a final volume of 20 μL. The PCR primers were designed using Primer Premier 5.0. The thermal cycle conditions were as follows: 2 min at 50°C and then 95°C for 10 min, followed by 40 cycles of 95°C for 15 s and 60°C for 1 min. The uniform amplification of the products was confirmed by analyzing the melting curves of the amplified products. GAPDH was used as an endogenous control for each sample. The primers used for each of the genes are listed in S1 Table.

### Western blotting

Western blot was performed as previously described [20]. The anti-S100A7 monoclonal antibody was purchased from Abcam (No. ab13680). The target proteins were detected using either a chemiluminescent substrate or DAB. β-Actin was used as an internal control for equal protein loading.

### Immunohistochemistry

Immunohistochemical staining for S100A7 in the tissue arrays was performed as previously described [21]. Keratin-13 tissue staining was performed as for S100A7. Cell immunostaining of S100A7 and keratin-13 was performed as follows. First, the cells were plated on cover-glass and cultured for different numbers of days and fixed with 4% buffered paraformaldehyde after washing with PBS. The cells were then permeabilized with 0.5% Triton X-100 for 10 min at room temperature. The subsequent steps were performed as previously described [21]. Isotype IgG was used as a negative control. Two pathologists evaluated all tissue immunostaining for S100A7 independently. Any staining in the cytoplasm, nucleus, or cytoplasmic membrane was considered positive. The cancers were scored using our previously reported scale [20]: 0, no positive cells; 1, <10% cancer cells positive; 2, >10% and <50% cancer cells positive; 3, >50% and <75% cancer cells positive. Grade 1: well-differentiated; Grade 2: moderately-differentiated; Grade 3: poorly differentiated. To calculate S100A7-positive cells in suspension, cells were cultured in suspension for 48 hours and then reseeded on the glass-cover slides and cultured for 12 hours,and finally examined by immunohistochemistry with specific anti-S100A7 antibody and control antibody, respectively. S100A7-positive and–negative cells were counted in each clump of cells under a microscope. The average value of S100A7-positve in 20 clumps of cells for each cell lines were used.

### Double immunofluorescence staining

Double immunofluorescence labeling of S100A7 and keratin-13 was performed. First, the cells were plated, fixed, and permeabilized as for the cell immunostaining. The cells were then blocked with 3% BSA-PBS solution. S100A7 antibody was applied for 1 h at 37°C followed by washing and subsequent incubation with TRITC-labeled goat anti-rabbit IgG for 1 h at 37°C. The cells were blocked again and incubated with keratin-13 antibody (mouse monoclonal IgG), and FITC-labeled goat anti-mouse IgG was used to detect keratin-13 expression. Finally, the nuclei were stained using DAPI, and S100A7 and keratin-13 expression was detected using a fluorescence microscope.

### siRNA interference

To knockdown the expression of S100A7, small interfering RNAs were purchased from Gene Pharma. The targeted sequences were as follows: CCAGACGUGAUGACAAGAUTT and AUCUUGUCAUCACGUCUGGTT. The siRNA was designed according to the sequence provided under GenBank accession number NM_002963.3 S100A7. Scrambled siRNA was used as the control. HCC94 cells were transfected with S100A7-siRNA and scrambled siRNA using the Lipofectamine 2000 Transfection Reagent according to the manufacturer's protocol.

### S100A7 over-expression vector construction and transfection

To construct the expression plasmid for the S100A7 protein, the S100A7 cDNA fragment was amplified using the primers5’-CCGCTCGAGATGAGCAACACTCAAGCTGA-3’ and 5’-CGGAATTCTCACTGGCTGCCCCCGGAAC-3’. The amplified cDNA was inserted into the pcDNA3.1+ vector. The cells were transfected with the plasmids using the Lipofectamine 2000 Transfection Reagent, and empty vector was used as the control. For the generation of stable transfectants, A-431 cells were transfected with pcDNA3.1/S100A7, and pcDNA3.1 empty plasmids. After 2 weeks G418 (400μg/mL) treatment, G418-resistant clones were selected, and those clones were then expanded for further studies.

### Tumorigenicity in nude mice

Viable cells (5×10^6^) cells were mixed with HBSS and inoculated subcutaneously in six-week-old female nude mice. BALB/C nude (nu/nu) mice were bought from the Peking University Laboratory Animal Center (Beijing, PR China). Tumor growth was monitored as described in our previous study [22]. The tumors were washed with PBS and fixed with 4% buffered paraformaldehyde. All animal work was approved by Ethics Committee of College of Life Science, Beijing Normal University (CLS-EAW-2013-08). All methods were carried out in accordance with relevant guidelines by above Ethics Committee.

### Cell proliferation and survival rate in suspension

Cell proliferation and survival rate were evaluated by MTT and XTT [23–24]. The survival rates are expressed as a percentage of the absorbance reading of the control cells (% survival), and each condition was tested in six replicates.

### Statistical analysis

The statistical significance was evaluated using Student’s t-test (two-tailed) for comparisons between the experimental groups and the corresponding control groups. The differences were considered statistically significant at p less than 0.05.

## Results

### S100A7 is heterogeneously expressed in both squamous cell carcinoma tissues and cells

**Fig 1:**
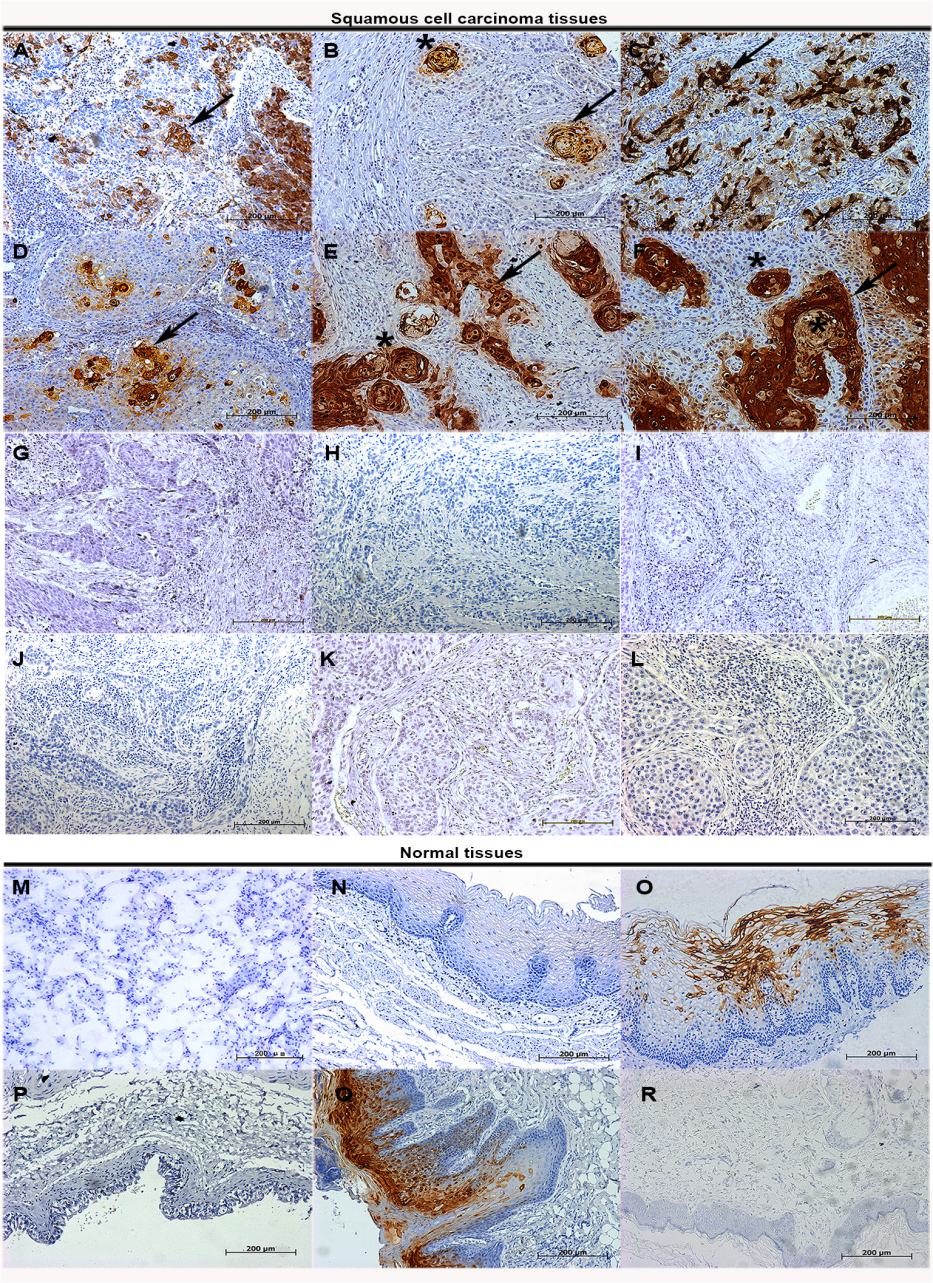
The expression of S100A7 in SCC tissues. SCC and normal tissues were examined by immunohistochemistry with specific anti-S100A7 antibody in lung (A, M); esophagus (B, N); cervix (C, O); bladder (D, P); oral cavity (E, Q); skin (F, R). The corresponding types of SCC tissues were also examined by immunohistochemistry with nonspecific IgG (G-L). Arrowheads indicate the positive staining of S100A7 and the asterisks indicate the keratinizing areas.

**Fig 2:**
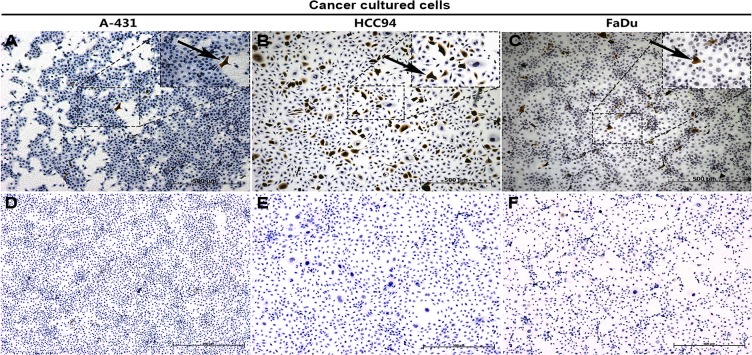
The expression of S100A7 in SCC cells. Cancer cells were examined by immunohistochemistry with specific anti-S100A7 antibody (A-C) and nonspecific IgG (D-F).

In our previous study, we reported that S100A7 was selectively expressed in lung SCC tissue specimens [20–21]. To determine whether a similar expression of S100A7 is present in the other SCCs, six types of SCCs tissue arrays with 452 cases of SCCs were examined for S100A7 expressions using immunohistochemistry. The results showed that 41% of lung SCCs, 76.6% of esophageal SCCs, 50.6% of cervical SCCs, 80.4% of oral SCCs, 84.8% of skin SCCs and 66.7% of bladder SCCs were positive for S100A7 (Table 1). Importantly, we found that S100A7 expression was heterogeneous and displayed a patchy or a scattered distribution in all positively stained tissues, and the staining intensity of S100A7 was inversely associated with degree of differentiation in all tested SCC tissues Representative immunoreactivity results are shown in Fig 1A–1F). In addition, S100A7 expression was also found in the normal tissues including cervix and oral cavity, but was absent from normal lung, bladder, esophagus tissues, and skin (Fig 1M–1R). Tissue section using nonspecific IgG as negative control showed no positive staining (Fig 1G–1L). Based on the characteristics of S100A7 expression in SCC tissues, we hypothesized that S100A7 heterogeneity may exist in vitro. To test our hypothesis, three squamous carcinoma cell lines, including HCC94, FaDu, and A-431 cells, were selected and the S100A7 expression was examined by immunohistochemistry. Unlike with HCC94 cells, less than 1% S100A7-positive cells were detected in FaDu and A-431 cells in the normal culture conditions (Fig 2A–2C). Cultured cells using nonspecific IgG as negative control showed no positive staining (Fig 2D–2F). These results suggest that the heterogeneity of S100A7 is a common feature in both SCC tissues and cultured SCC cells.

**Table 1:** S100A7 expression in squamous cell carcinoma tissues.

			S100A7 IHC staining score						
Group	Differentiation degree	No.tested	0	1	2	3	4	No.Positive	Percentage of positive(No.positive/No.tested)x100%
Oral SCC samples	Grade1-2	44	6	10	14	5	9	38	86
	Grade3	5	3	1	1	0	0	2	40
	Unidentified	2	1	1	0	0	0	1	50
Skin SCC samples	Grade1-2	144	13	25	41	42	23	131	91
	Grade3	8	6	1	0	1	0	2	25
	Unidentified	6	5	0	0	0	1	1	17
Esophageal SCC samples	Grade1-2	38	0	18	12	7	1	38	100
	Grade3	52	21	22	8	0	1	31	60
Lung SCC samples	Grade1-2	25	3	13	5	1	3	22	88
	Grade3	50	41	3	5	0	1	9	18
Cervical SCC samples	Grade1-2	42	15	11	10	3	3	27	64
	Grade3	32	21	3	6	1	1	11	34
	Unidentified	1	1	0	0	0	0	0	0
Bladder SCC samples	Grade1-2	3	1	1	0	1	0	2	67

Abbreviation: SCC, squamous cell carcinomas

### S100A7-negative cells become positive in confluent culture

**Fig 3:**
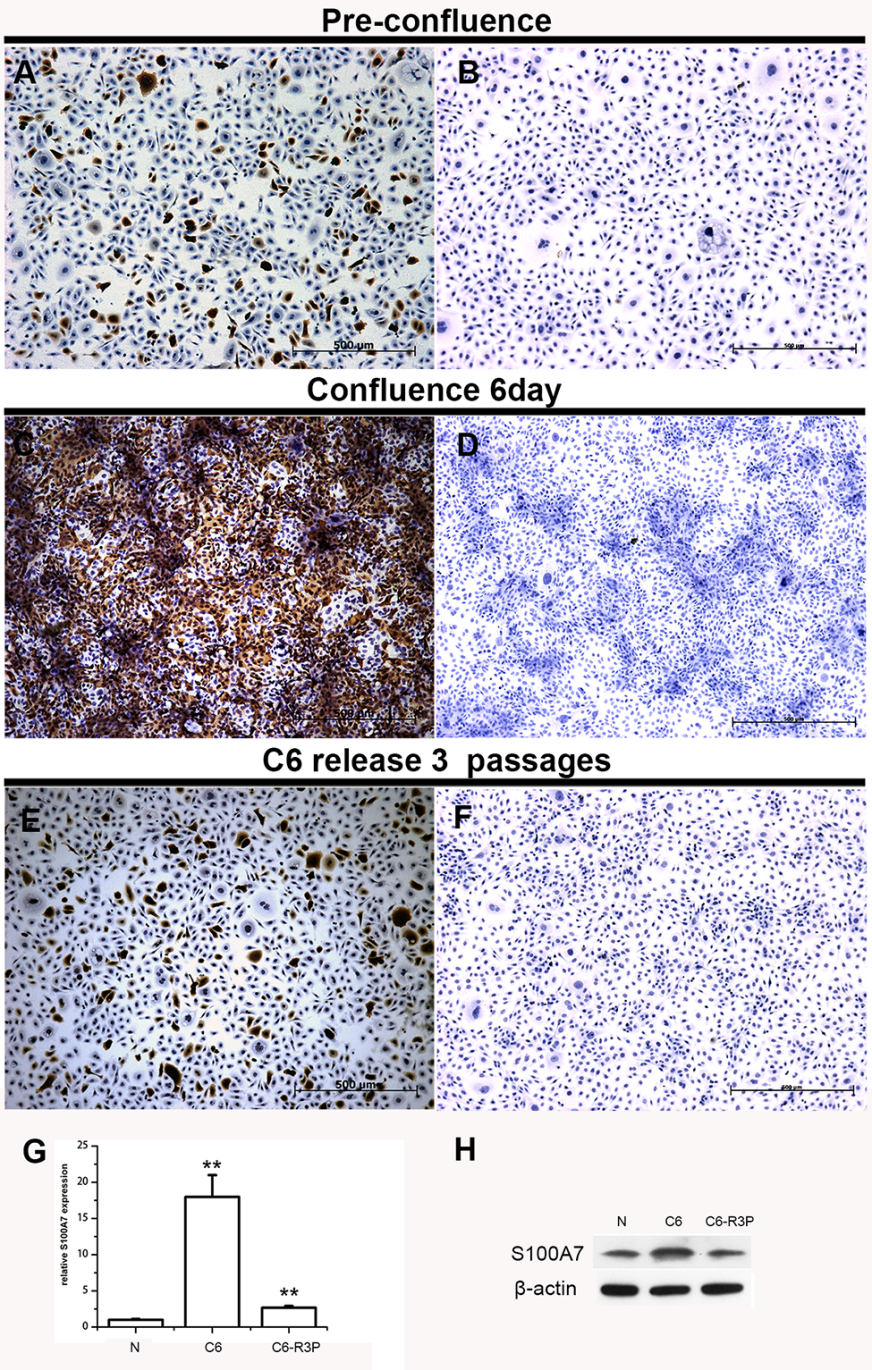
The induction of S100A7 expression in HCC94 cells in confluent culture. S100A7 expression was examined by immunohistochemistry (A, C, E), real-time PCR (G) and Western blotting (H). Cells were also examined by immunohistochemistry with nonspecific IgG (B, D, F).*, p<0.05, and **, p<0.01 versus the experimental groups and the pre-confluent groups. The results represent the average values from three independent experiments. N: pre-confluence; C6: cells were cultured at confluence for 6 days; C6-R3P: cells that were cultured at confluence for six days and then reseeded at sub-confluence for 3 cell passages.

The inconsistent proportions of S100A7-positive cells in vitro and in vivo in SCCs suggest that there is a factor that triggers this difference. It has been reported that S100A7 expression could be induced in HEKn cells under confluent culture [19]. To investigate whether confluent culture also induces S100A7 expression in SCC cells, HCC94, A-431, and FaDu cells were cultured under confluent conditions. Unfortunately, A-431 and FaDu cells were easily detached in confluent culture, only HCC94 cells were successfully to culture under the confluence for 6 days. Notably, the percentage of S100A7-positive cells was increased from about 10% under the normal culture condition to above 80% under confluence conditions, and then significantly decreased after the confluent cells were reseeded at the pre-confluent density and returned to the original ratio after three cell passages. Although the percentage of S100A7-positive cells was significantly different before and after the confluent culture, the subcellular localization and intensity of the S100A7 immunostaining were the same (Fig 3A, 3C and 3E). In contrast, cultured cells using nonspecific IgG as negative control showed no positive staining (Fig 3B, 3D and 3F). As expected, the expression of both S100A7 mRNA and proteins also changed based on the increased or decreased percentage of S100A7-positive cells (Fig 3G and 3H). Collectively, these results indicate that S100A7-positive cells could be inducible in HCC94 in the confluent culture and S100A7-negative and–positive cells bi-directionally convert to each other, depending on the cell density.

### S100A7-negative cells convert into positive cells in suspension culture

**Fig 4:**
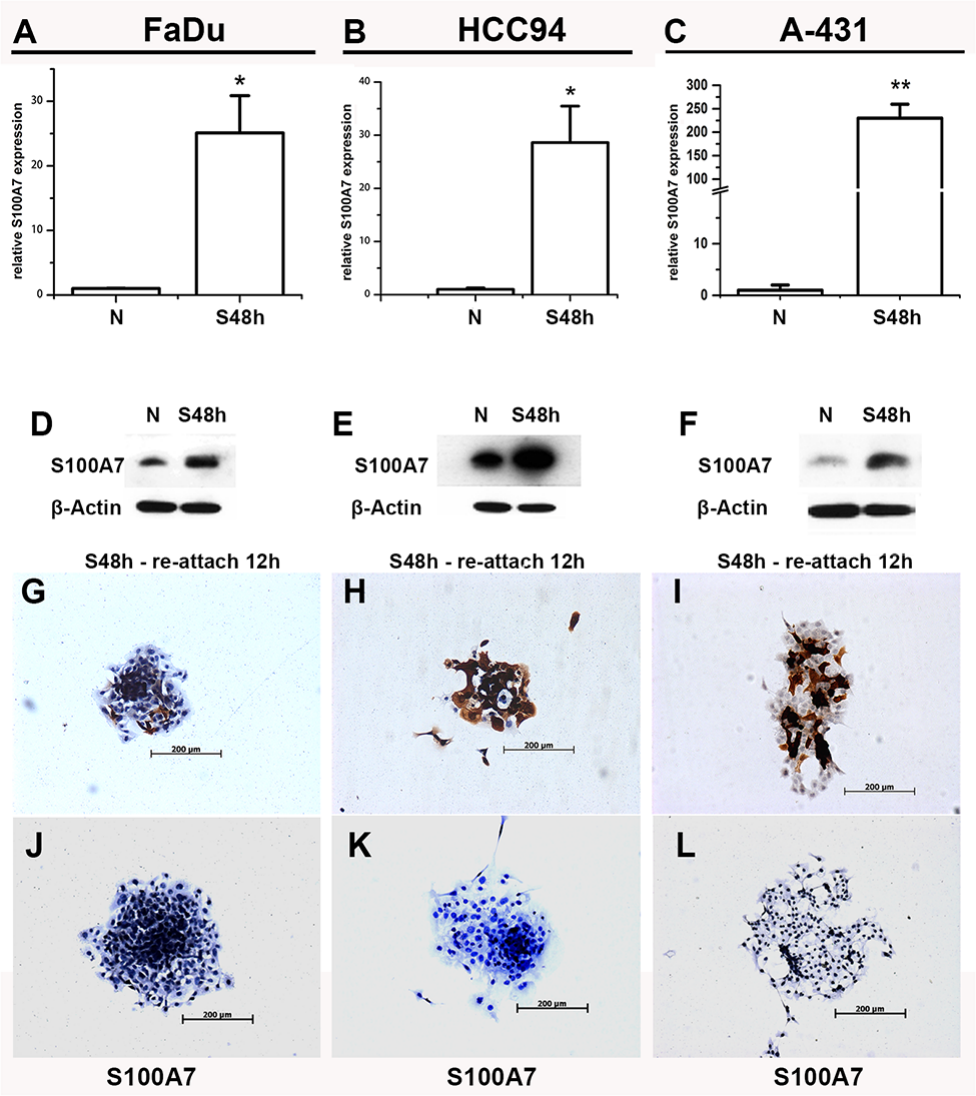
The induction of S100A7 expression in FaDu, A-431, and HCC94 cells in suspension culture. The expression of S100A7 was determined by real-time PCR (A-C) and Western blotting (D-F). FaDu, HCC94 and A-431cells in suspension for two days were reattached for 12h and examined using immunohistochemistry with specific anti-S100A7 antibody (G-I) and nonspecific IgG (J-L). S48h: suspension 48h. N: pre-suspension.*, p<0.05, and **, p<0.01 versus the suspension groups and the pre-suspension groups. The results represent the average values from three independent experiments.

Suspension culture is another inducer of S100A7 expression in keratinocytes [19]. To explore whether S100A7 may also be induced by suspension in SCC cells, HCC94, FaDu, and A-431 cells were cultured in suspension for two days. Similar to the results of the confluence experiment, the expression of S100A7 was induced by suspension in all three SCC cells (Fig 4A–4F). To further confirm whether the elevated levels of S100A7 expression resulted from an increased percentage of S100A7-positive cells, the suspension cells were reattached to a slide for 12 h and then examined by immunohistochemistry. As expected, the percentage of S100A7-positive cells was raised from less than 1% in FaDu and A-431 cells, and about 10% in HCC94 cells in the normal culture to above 30%, 50% and 80% after suspension treatment, respectively; the induced-positive cells also displayed heterogeneous expression (Fig 4G–4I), indicating that the S100A7-positive cells are also inducible by suspension. Cultured cells using nonspecific IgG as negative control showed no positive staining (Fig 4J–4L).

### S100A7-positive cells are inducible in vivo

**Fig 5:**
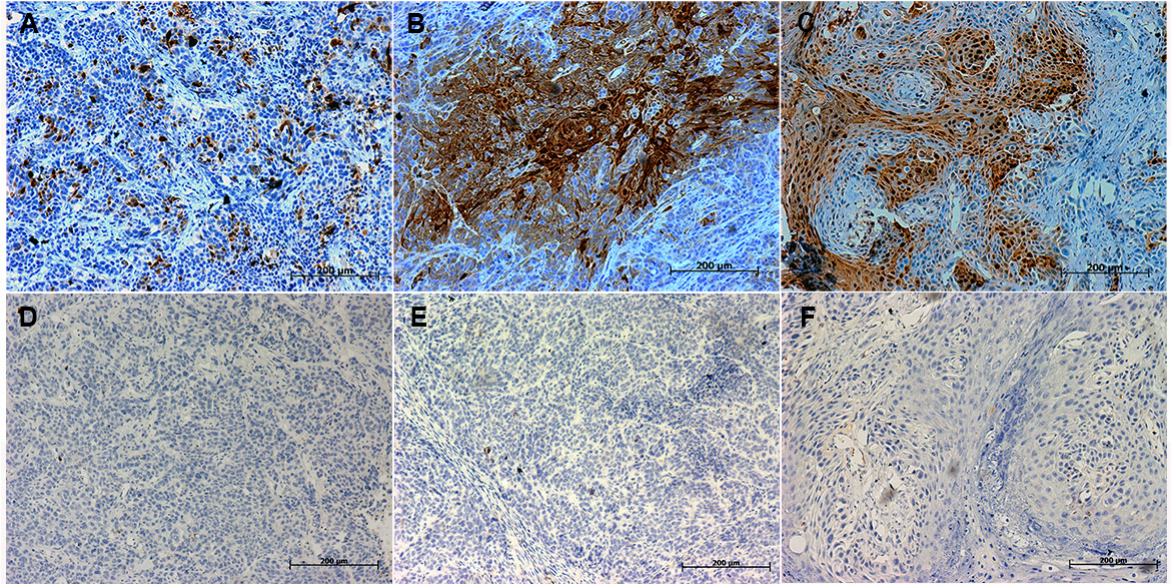
Immunohistochemical analysis of S100A7 protein expression in xenografts. S100A7 expression was examined in FaDu xenografts, A-431 xenografts, and HCC94 xenografts by immunohistochemistry with specific anti-S100A7 antibody (A-C) and nonspecific IgG (D-E).

To determine whether S100A7 induction in SCC cells occurs in vivo, we examined S100A7 expression in xenografts derived from FaDu, A-431, and HCC94 cells using immunohistochemistry. Similar to the distribution of S100A7 staining in specimens from SCC patients, S100A7-positive cells displayed patchy or scattered pattern, and marked heterogeneity of S100A7 staining existed in all tested xenografts. Importantly, the percentage of S100A7-positive cells was dramatically raised in their corresponding xenografts, respectively (Fig 5A–5C). In contrast, tissue sections using nonspecific IgG as negative control showed no positive staining (Fig 5D–5F). These results directly support the hypothesis that S100A7 induction in SCC cells also occurs in vivo.

### Squamous differentiation markers are induced in SCC cells both in vitro and in vivo

**Fig 6:**
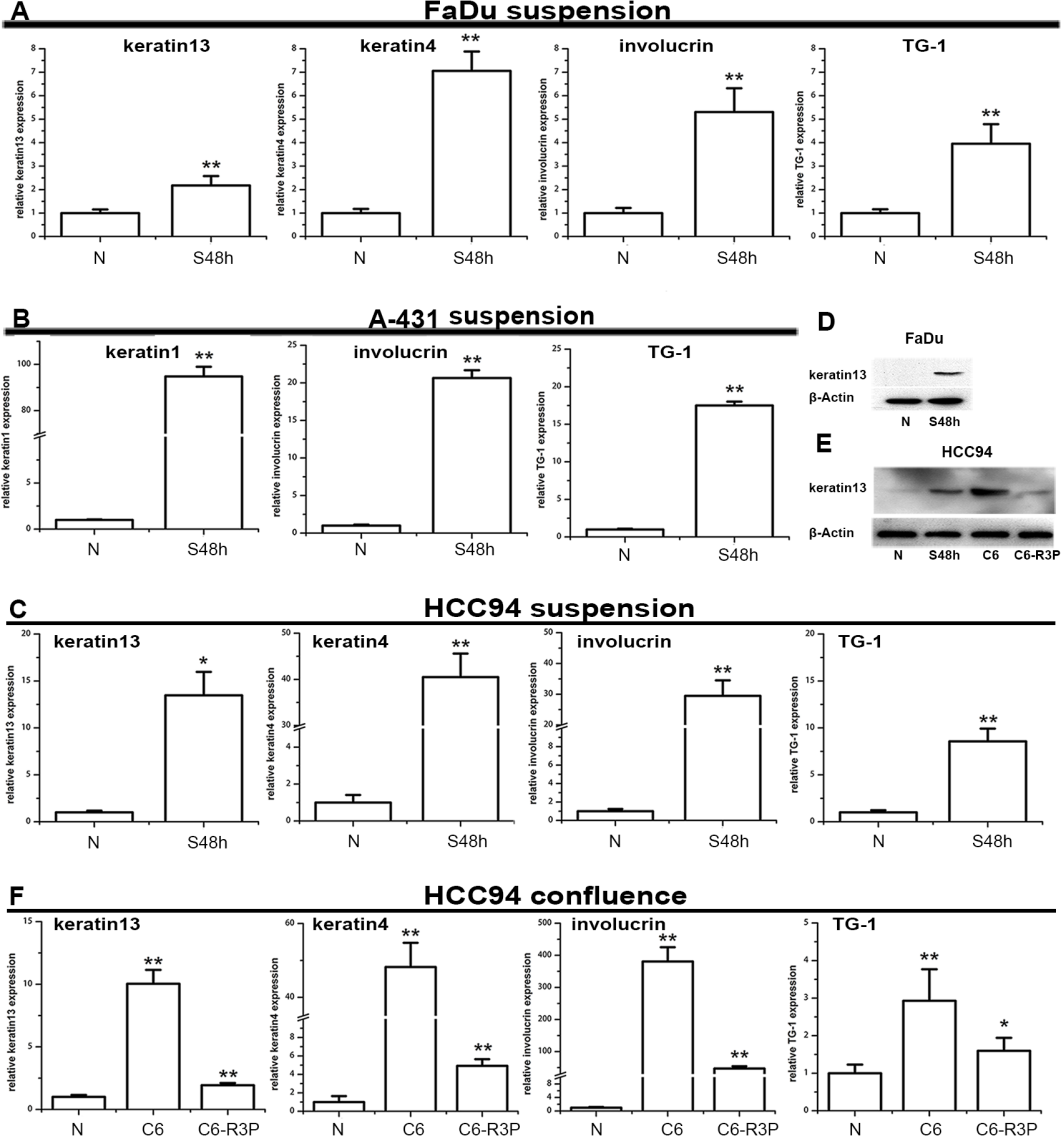
Squamous differentiation markers were also induced by suspension and confluent treatment. The expression of keratin-1, keratin-13, keratin-4, TG-1 and involucrin was determined by real-time PCR for the FaDu (A); A-431(B) and HCC94 (C, F) cells after suspension or confluent culture. The protein level of keratin-13 was confirmed by Western blotting for the FaDu (D) and HCC94 (E) cells. S48h: suspension 48h. N: pre-suspension. C6: cells were cultured at confluence for six days. C6-R3P: cells were cultured at confluence for six days and then reseeded at sub-confluence for 3 cell passages. *, p<0.05, and **, p<0.01 versus the S100A7 experiential groups and the control groups. The results represent the average values from three independent experiments.

**Fig 7:**
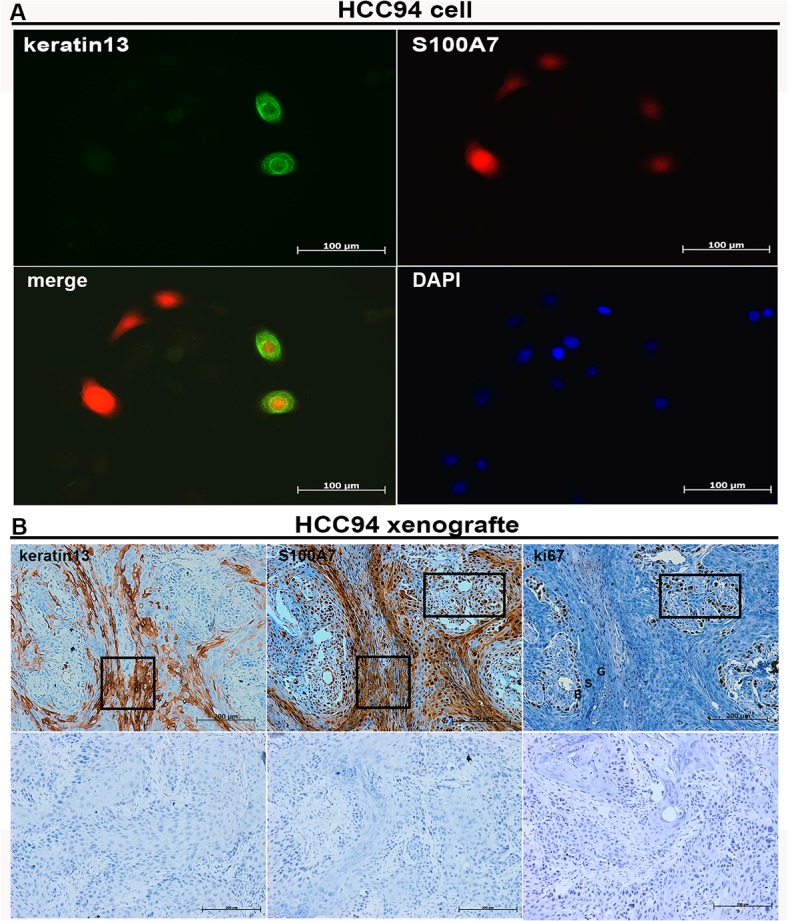
The relationship of S100A7 and keratin-13 expression was examined by immunofluorescence and by immunohistochemistry. The expression of keratin-13 and S100A7 in HCC94 cells was examined by immunofluorescence (A) and in HCC94 xenografts (B) by immunohistochemistry with specific antibody (B, Top panel) in the consecutive sections, and nonspecific IgG (B, Bottom panel). Box areas indicate the positive staining of keratin 13, S100A7 and Ki67 in the basal and suprabasal layers of xenograft. ‘B’ indicates stratum basale; ‘S’ indicates stratum spinosum; ‘G’ indicates stratum granulosum.

It has been reported that SCCs could express the keratins pattern shown by their normal epithelium, such as K4/K13 in non-keratinizing epithelia and K1/K10 in keratinizing epithelia [25]. Our novel findings were that the expression of differentiation markers including keratin-1, keratin-4, keratin-13, TG-1 and involucrin was also significantly increased in A-431, FaDu, and HCC94 cells after suspension and/or the confluent culture, respectively (Fig 6A–6F). However, no expression of keratin-10 and keratin-4 was detected in A-431 cells and no difference expression of keratin-13 was observed in A-431 cells cultured in the normal condition and suspension (S1 Fig). Given that S100A7 and differentiation-specific markers were induced in vitro by same stimuli in the above mentioned SCC cell lines, we wished to know the expression pattern of these proteins in vitro and in vivo. Based on the aforementioned results, we selected keratin-13 to as representative differentiation markers among others. For one reason, keratin-13 could be detected in both HCC94 cells and FaDu cells, whereas, keratin-1 only in A-431 cells but not in HCC94 and FaDu cells. The other reason is that HCC94 xenografts display a distinct squamous differentiation phenotype due to its being a well-differentiated cervical squamous carcinoma cell line. The results revealed that some S100A7-positive cells displayed co-localization with keratin-13-positive cells, but others were not (Fig 7A). In HCC94 xenograft, a distinct squamous differentiation phenotype, with clear stratum basale, stratum spinosum and stratum granulosum was found because HCC94 is a well-differentiated cervical squamous carcinoma cell line and becomes well-differentiated in vivo. Notably, S100A7-positive staining in the stratum spinosum and stratum granulosum was distributed in the both cytoplasm and/or nucleus, but in the stratum basale, staining occurred only in the nucleus. Keratin-13 demonstrated a similar staining pattern in the suprabasal layer where S100A7-positive staining was found in both the cytoplasm and the nucleus, whereas keratin-13 was only detected in the cytoplasm. Importantly, some S100A7-positive cells with only nuclear staining co-expressed with Ki67 in the basal layer. In contrast, tissue sections using nonspecific IgG as negative control showed no positive staining (Fig 7).

### S100A7 promotes squamous cell carcinoma proliferation and survival and inhibits squamous cell differentiation

**Fig 8:**
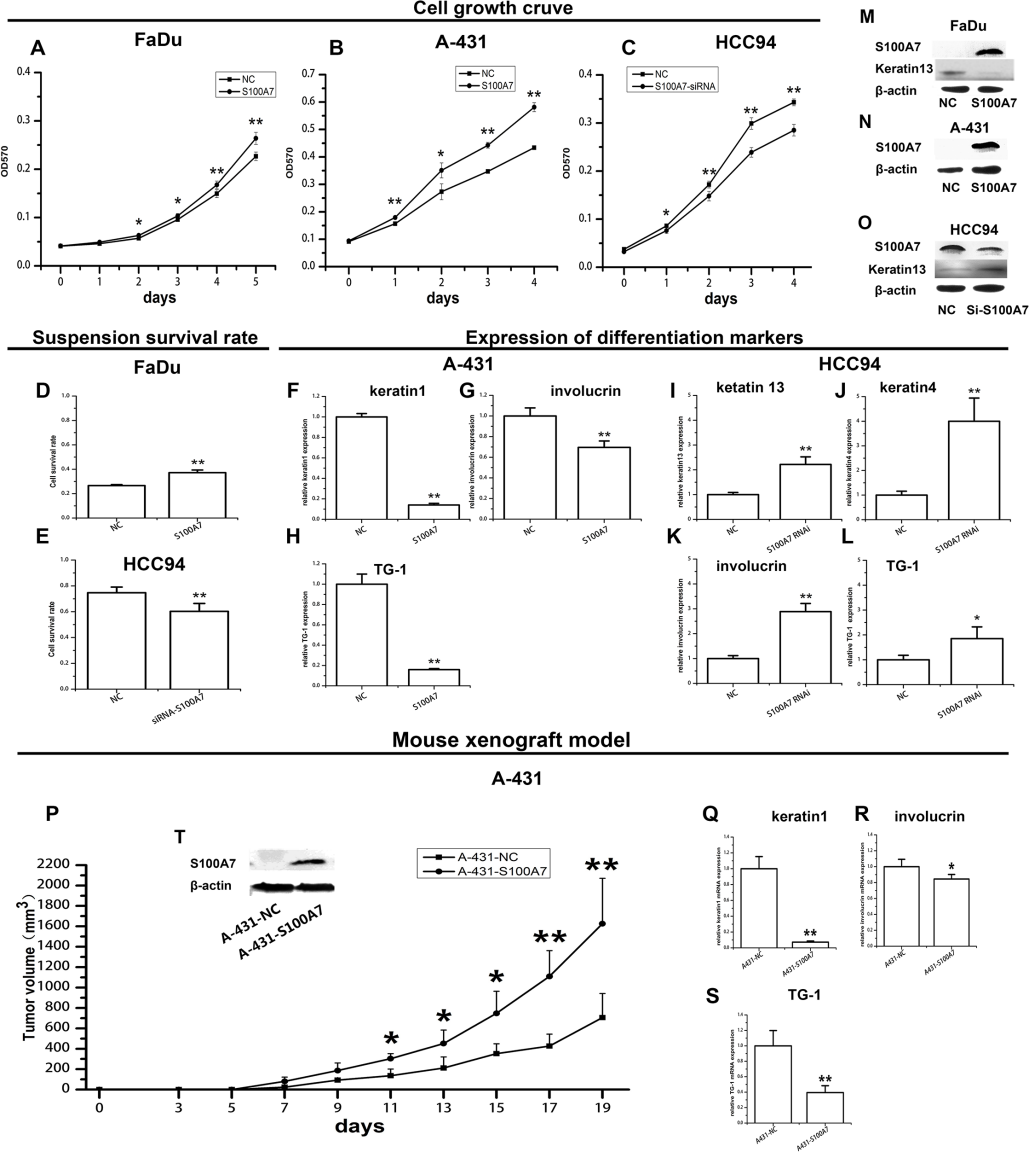
S100A7 promotes squamous carcinoma cell growth and inhibits squamous cells differentiation both in vitro and in vivo. The proliferation and the survival rate of SCC cells were evaluated by MTT (A, B, C) and XTT (D, E). The expression of differentiation markers was determined by real-time PCR (F-L). The expressions of S100A7 and keratin-13 after overexpression or knockdown S100A7 in SCC cells were confirmed by Western blotting (M, N, O). ‘NC’ stands for mock-transfected cells. The volumes of tumor xenografts derived from the stable-overexpressing S100A7-A431 cells and the control cells (P). The expression of differentiation markers in xenografts was examined by real-time PCR (Q-S). The stable-overexpressing S100A7 in A-431 cells was confirmed by Western blotting (T). *, p<0.05, and **, p<0.01 versus the S100A7 experiential groups and the control groups. The results represent the average values from six independent experiments.

The above findings that the expression of S100A7 can be induced in SCC cells by suspension and confluent culture seem to imply that S100A7 upregulation may involve in SCC cell differentiation. To explore the roles of S100A7 in SCCs, S100A7 knockdown or overexpression was performed in HCC94, FaDu, and A-431 cells, depending on the endogenous levels of S100A7. We found that the cell proliferation and survival rate of the FaDu and A-431 cells were significantly increased in the S100A7 overexpressed group compared to the control group (Fig 8A, 8B and 8D). Converse results were obtained in the HCC94 cells with silencing of S100A7 (Fig 8C and 8E). In parallel, the expression of the differentiation-specific markers keratin-4, keratin-13, TG-1, and involucrin were significantly increased by S100A7 knockdown and decreased by S100A7 overexpression (Fig 7F–7L). Silencing or overexpression of S100A7 and the expression of keratin-13 were also confirmed by Western blot (Fig 8M, 8N and 8O). To gain further insight into the function of S100A7 in vivo, the stable-overexpressing S100A7-A431 cells and control cells were implanted into the left and right flank areas of nude mice. Similar results were also obtained in vivo. The overexpression of S100A7 significantly promoted tumor growth and decreased the expression of cytokeratin-1, TG-1, and involucrin in A-431 xenografts compared with the control animals (Fig 8P–8S). Taken together the above results indicate that S100A7 expression promotes SCC cell proliferation and inhibits cell differentiation in the tested cell lines.

## Discussion

In the present study, we examined the expression of S100A7 in 452 SCC specimens, including the lung, esophagus, bladder, cervix, oral cavity, and skin. The results demonstrated that S100A7 expression occurred in the majority of SCC tissues, with stronger staining in well-differentiated tissues or well-differentiated areas within one section. In addition, this is the first report that S100A7 is also expressed in SCCs of the esophagus and cervix. These results strongly suggest that S100A7 may be a common marker of SCCs. Further examination found that the heterogeneity of S100A7 existed in all S100A7-positive specimens, and S100A7 appeared in a patchy or scattered distribution. Similarly, the heterogeneity of S100A7 was also detected in cultured A-431, FaDu, and HCC94 cells and in their corresponding xenografts. The heterogeneity of the S100A7 in the SCC cells and SCC tissues may be because of the origin of the SCCs, which arise from multilayered epithelia that undergo continuous self-renewal in a basal layer and differentiate to a superficial direction under normal conditions [26]. Therefore, the heterogeneity of S100A7 may be caused by the cellular heterogeneity within SCC tissues or cultured cells.

Interestingly, we found that the expression of S100A7 was dramatically induced in all tested SCC cell lines both in vitro and in vivo. We suggest two reasons for this difference. First, is the proliferation of S100A7-positive cells in vivo, and second, is S100A7 induction in vivo. In support of this hypothesis are our results showing that S100A7-positive cells are significantly induced by confluent and suspension cultures. Furthermore, S100A7-positive cells also become negative cells when the inducer is removed. These results indicate that S100A7-negative and–positive cells might directionally convert to each other depending on the microenvironment, such as cell density or suspension. In addition, we also found that S100A7-positive cells could generate the same positive cells only for limited cell passages in vitro (data not shown). Taken together, our results indicate that S100A7-positive cells are induced both in vitro and in vivo, either by proliferation or induction or both.

Although S100A7 and differentiation-specific markers both were inducible expression by confluent and suspension cultures, even in vivo, we found that cell proliferation, survival rate, and/or tumor growth were significantly increased in S100A7 overexpressing cells, whereas the expression differentiation-specific markers was markedly decreased. Conversely, inhibition of cell proliferation and upregulation of differentiation markers were detected by silencing of S100A7. These results indicate that both endogenous and ectopic expression of S100A7 plays the similar role in promoting the growth and inhibiting the differentiation of squamous cancer cells, at least for our tested SCC cells. In addition, overexpressing S100A7 in A-431 cells could dramatically inhibit the induction of differentiation markers by suspension (data not shown). Our unpublished results demonstrated that S100A7 acts as a dual regulator in promoting proliferation and suppressing squamous differentiation through the GATA-3/caspase-14 pathway in A-431 cells. On the other hand, S100A8 and S100A9, two members of the S100 family, display the similar expression and function in SCC. It has been reported that the expression of S100A8/A9 was positively correlated with differentiation in clinical SCC tissues. However, in SCC12 cells, overexpressing S100A8 and/or S100A9 had increased proliferation and cell migration capacity in vitro and also showed increased tumor growth in vivo in SCID mice [27]. Therefore, we conclude that the expression and function of S100A7 are highly cell-type-specific and its functions range from regulating cell proliferation and differentiation.

In the present study, we have demonstrated that S100A7 is a common marker of squamous cell carcinomas and displays the cellular heterogeneity in SCC tissues and cells. In addition, S100A7-positive cells could be inducible in SCC cell lines both in vitro and in vivo depending on the microenvironments. S100A7 acts as a dual regulator in promoting cell growth and inhibiting cell differentiation. Further elucidation of the molecular mechanisms of S100A7 induction and its exact role in cell differentiation are ongoing in our laboratory.

No potential conflicts of interest were disclosed.

## Supporting Information

S1 Fig The expression of keratin-4 and keratin-13 in A-431 cells. (TIF)

S1 Table Primers used for each of the genes. (DOC)
