# Supplementary figures and images for: The Characteristics and Function of S100A7 Induction in Squamous Cell Carcinoma: Heterogeneity, Promotion of Cell Proliferation and Suppression of Differentiation
Source: PLoS One. 2015 Jun 8;10(6):e0128887. doi: 10.1371/journal.pone.0128887 (PMC4460013; doi:10.1371/journal.pone.0128887)

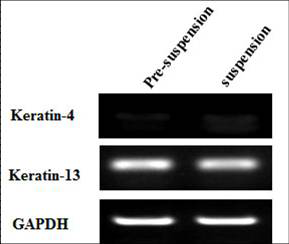

Supplement: S1 Fig — (TIF) [file pone.0128887.s001.tif]
