# Supplementary material for: The Characteristics and Function of S100A7 Induction in Squamous Cell Carcinoma: Heterogeneity, Promotion of Cell Proliferation and Suppression of Differentiation
Source: PLoS One. 2015 Jun 8;10(6):e0128887. doi: 10.1371/journal.pone.0128887 (PMC4460013; doi:10.1371/journal.pone.0128887)
Supplement: S1 Table — (DOC) [file pone.0128887.s002.doc]

S1 Table Primers used for each of the genes

| S100A7-sense | 5’CTTCCCCAACTTCCTTAGTG3’ |
| --- | --- |
| S100A7-antisense | 5’GTAGTCTGTGGCTATGTCTC3’ |
| Keratin13-sense | 5’TATGGAGGCGGCGTGAGC3’ |
| Keratin13-antisense | 5’CCAGGCGGTCGTTGAGGTT3’ |
| Keratin4-sense | 5’TTAAAGATGCCCACAGCAAGCG3’ |
| Keratin4-antisense | 5’GGTGGCGATCTCGATGTCCAA3’ |
| Involucrin-sense | 5’CAGCCAACTCCACTGCCTCCC3’ |
| Involucrin-antisense | 5’CTGCTCCTGTGGCTCCTTCTGC3’ |
| TG-1-sense | 5’CCGTGGAGACCCAGTCAAT3’ |
| TG-1-antisense | 5’CGGAATATCCCGTGCGTAG3’ |
| Keratin-1-sense | 5’TTATGGTCCTGTCTGCCCTCC3’ |
| Keratin-1-antisense | 5’ TGACTTGATTTGCTCCCTTTC 3’ |
| GAPDH-sense | 5’ GAGTCAACGGATTTGGTCGT 3’ |
| GAPDH-antisense | 5’ GACAAGCTTCCCGTTCTCAG3’ |
